# An analysis of key indicators of reproducibility in radiology

**DOI:** 10.1186/s13244-020-00870-x

**Published:** 2020-05-11

**Authors:** Bryan D. Wright, Nam Vo, Johnny Nolan, Austin L. Johnson, Tyler Braaten, Daniel Tritz, Matt Vassar

**Affiliations:** 1 Oklahoma State University Center for Health Sciences, 1111 W 17th St, Tulsa, OK 74107 USA; 2 Kansas City University of Medicine and Biosciences, Joplin, MO USA; 3 Department of Diagnostic and Interventional Imaging, The University of Texas Health Sciences Center at Houston, Houston, TX USA

**Keywords:** Meta-analysis, Reproducibility of results, Radiology, Transparency

## Abstract

**Background:**

Given the central role of radiology in patient care, it is important that radiological research is grounded in reproducible science. It is unclear whether there is a lack of reproducibility or transparency in radiologic research.

**Purpose:**

To analyze published radiology literature for the presence or lack of key indicators of reproducibility.

**Methods:**

This cross-sectional retrospective study was performed by conducting a search of the National Library of Medicine (NLM) for publications contained within journals in the field of radiology. Our inclusion criteria were being MEDLINE indexed, written in English, and published from January 1, 2014, to December 31, 2018. We randomly sampled 300 publications for this study. A pilot-tested Google form was used to record information from the publications regarding indicators of reproducibility. Following peer-review, we extracted data from an additional 200 publications in an attempt to reproduce our initial results. The additional 200 publications were selected from the list of initially randomized publications.

**Results:**

Our initial search returned 295,543 records, from which 300 were randomly selected for analysis. Of these 300 records, 294 met inclusion criteria and 6 did not. Among the empirical publications, 5.6% (11/195, [3.0–8.3]) contained a data availability statement, 0.51% (1/195) provided clear documented raw data, 12.0% (23/191, [8.4–15.7]) provided a materials availability statement, 0% provided analysis scripts, 4.1% (8/195, [1.9–6.3]) provided a pre-registration statement, 2.1% (4/195, [0.4–3.7]) provided a protocol statement, and 3.6% (7/195, [1.5–5.7]) were pre-registered. The validation study of the 5 key indicators of reproducibility—availability of data, materials, protocols, analysis scripts, and pre-registration—resulted in 2 indicators (availability of protocols and analysis scripts) being reproduced, as they fell within the 95% confidence intervals for the proportions from the original sample. However, materials’ availability and pre-registration proportions from the validation sample were lower than what was found in the original sample.

**Conclusion:**

Our findings demonstrate key indicators of reproducibility are missing in the field of radiology. Thus, the ability to reproduce studies contained in radiology publications may be problematic and may have potential clinical implications.

## Key points

- Key indicators of reproducibility and transparency are frequently missing in the radiology literature.
- The ability to reproduce the results of radiologic studies may be difficult.

## Introduction

The field of radiology plays a significant role in the diagnosis, monitoring, and treatment of numerous disease processes. The importance of radiology to the field of medicine is evident by the large amount of annual expenditures on imaging, estimated to be 10% of total healthcare costs in the USA [1]. Advancements in imaging modalities and diagnostic testing are predicated upon robust and trustworthy research. Yet, the field of radiology has been known for low-level evidence study designs, with randomized trials, multicenter studies, and meta-analyses making up the smallest portion of publications (0.8 to 1.5%) [2]. With the movement toward patient-centered, evidence-based care, efforts are needed to ensure the robustness and reproducibility of radiology research.

Reproducibility—defined as the ability to conduct an independent replication study and reach the same or similar conclusions as the study in question [3, 4]—gained national attention after the majority of 1500 surveyed scientists reported failure to reproduce another scientist’s experiment and half being unable to reproduce their own experiments [5]. In radiology research, a lack of reproducibility has been partly attributed to imaging datasets too small to power a significant finding, models lacking independent validation, and improper separation of training and validation data [6]. Such practices may go undetected by editors, peer reviewers, and readers and contribute to downstream effects, such as irreproducible results and perpetuated errors in subsequent studies.

Given the central role of radiology to patient care, reproducible radiology research is necessary. In this study, we investigate radiology publications for key factors of reproducibility and transparency. Findings may be used to evaluate the current climate of reproducible research practices in the field and contribute baseline data for future comparison studies.

## Materials and methods

Our investigation was designed as a cross-sectional meta-research study to evaluate specific indicators of reproducibility and transparency in radiology. The study methodology is a replication of work done by Hardwick et al. [7] with minor adjustments. Our analysis did not involve human subjects; thus, this investigation was not subject to institutional review board oversight [8]. Guidelines detailed by Murad and Wang were used for the reporting of our meta-research [9]. The Preferred Reporting for Systematic Reviews and Meta-Analyses (PRISMA) guidelines were used as necessary [10]. We supplied all protocols, raw data, and pertinent materials on the Open Science Framework (https://osf.io/n4yh5/). Amendments to our study, based upon peer review feedback following initial submission, are described in the protocol.

### Journal and study selection

One investigator (D.T.) searched PubMed with the NLM subject term tag “Radiology[ST]” on June 5, 2019. D.T. extracted the electronic ISSN number (or linking ISSN if the electronic version was unavailable) for included journals. PubMed was searched using the list of ISSN (PubMed contains the MEDLINE collection) on June 5, 2019, to identify publications. A random sample of 300 was selected to have data extracted with additional publications available as needed (https://osf.io/n4yh5/). With the goal of creating a diverse spectrum of publications for our study, restrictions were not placed on specific study types.

### Training

Three investigators (B.W., N.V., J.N.) underwent rigorous in-person training led by D.T. on data extraction and study methodology to ensure reliability between investigators. Training included a review of the following: objectives of the study, design of the study, protocol, Google form used for data extraction, and the process of extracting data. The process for extracting data was demonstrated via the use of 2 example publications. All investigators who underwent training independently conducted a blinded and duplicate extraction of data from 2 example publications. Once the mock data extraction was completed, the investigators (B.W., N.V., J.N.) convened and resolved any discrepancies present. The entire training session was recorded from the presenters’ point of view and was posted online for investigators to reference (https://osf.io/tf7nw/).

### Data extraction

Once all required training was completed, data were extracted from publications. Data extraction began on June 09, 2019, and was completed on June 20, 2019. One investigator (B.W.) performed data extraction on 300 publications with the other two investigators (N.V. and J.N.) extracting from 150 each. We divided publications into two categories: (1) publications with empirical data (e.g., clinical trial, cohort, case series, case reports, case-control, secondary analysis, chart review, commentary [with data analysis], and cross-sectional) and (2) publications without empirical data. For the sake of this study, imaging protocols with no patients or intervention were considered non-empirical. Different study designs resulted in a variation of data collected from individual publications. We analyzed non-empirical studies for the following characteristics: funding source(s), conflict of interest declarations, open access, and journal impact factor (dates, 5-year impact factor). Case reports and case series are not typically expected to be reproducible with a pre-specified protocol [11]. As a result, data were extracted from them in an identical manner as publications which lacked empirical data. There was no expectation for meta-analyses and systematic reviews to contain additional materials, meaning a materials’ availability indicator was excluded from their analysis. For the purpose of our study, data were synonymous with raw data and considered unaltered data directly collected from an instrument. Investigators were prompted by the data extraction form to identify the presence or absence of necessary pre-specified indicators of reproducibility and are available here *https://osf.io/3nfa5/*. The Google form implemented added additional options in comparison to the form Hardwick et al. [7] used. Our form had additional study designs such as case series, cohort, secondary analysis, meta-analysis/systematic review, chart review, and cross-sectional. Sources of funding were more specific to include non-profit, public, hospital, university, and private/industry. Following data extraction, all three investigators convened and resolved any discrepancies by consensus. Though unnecessary for this study, a third party was readily available for adjudication.

### Assessing open access

We systematically evaluated the accessibility of a full-text version of each publication. The Open-Access Button (*https://openaccessbutton.org/*) was used to perform a search using publication title, DOI, and/or PubMed ID. If search parameters resulted in an inaccessible article, Google Scholar and PubMed were searched using these parameters. If an investigator was still unable to locate a full-text publication, it was deemed inaccessible.

### Evaluation of replication and whether publications were cited in research synthesis

Web of Science was searched for all studies containing empirical data. Once located on Web of Science, we searched for the following: (1) the number of times a publication was used as part of a subsequent replication study and (2) the number of times a publication was cited in a systematic review/meta-analysis. Titles, abstracts, and full-text manuscripts available on the Web of Science were used to analyze if a study was cited in a systematic review/meta-analysis or a replication study.

### Reproducibility validation sample

Following peer-review, we extracted data from an additional 200 publications in an attempt to validate our results from the original 300. The additional 200 studies were selected from the list of initially randomized publications. The same authors (B.W., N.V., and J.N.) extracted data from the studies in a blind and duplicate manner identical to the original sample.

### Statistical analysis

Statistics from each category of our analysis were calculated using Microsoft Excel. Excel functions were used to provide quantitative analysis with results characterized by frequencies, percentages, and 95% confidence intervals using the Wilson Score for binomial proportions (*Dunnigan 2008*).

## Results

### Journal and publication selection

**Fig. 1:**
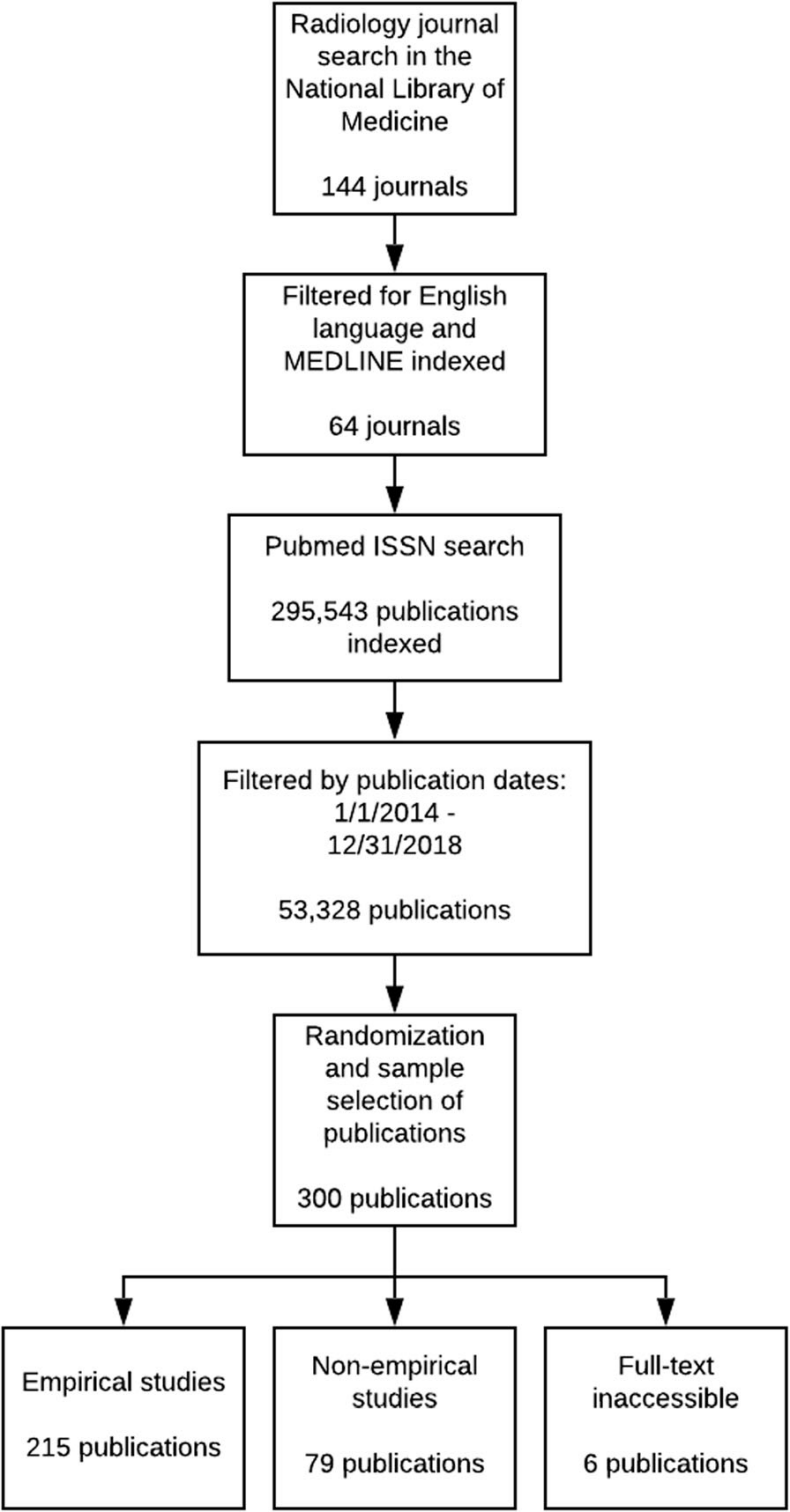
Publication selection process

The NLM catalog search identified 144 radiology journals, but only 64 met the inclusion criteria. Our PubMed search of journals identified 295,543 radiology publications with 53,328 being published within the time-frame. We randomly sampled 300, but only 294 publications were accessible. Of the eligible publications, 215 contained empirical data and 79 did not (Fig. 1). Publications without empirical data were excluded from select analyses because they could not be assessed for reproducibility characteristics. Furthermore, 20 publications were identified as either case studies or case series; these research designs are unable to be replicated and were excluded from the analysis of study characteristics. Study reproducibility characteristics were analyzed for 195 radiology publications (Table 1).

**Table 1:** Reproducibility indicators

*Reproducibility indicator*	*Role in producing transparent and reproducible science*	*Original sample (n* = 300)
**Articles**		
Article accessibility: articles were assessed for open accessibility, paywall access, or unable to access full text	Ease of access to publications enables interdisciplinary research by removing access barriers. Full-text access allows for validation through reproduction.	All (*n* = 300)
**Funding**		
Funding statement: presence of funding sources of the study	Funding provides researchers the ability to create new experiments and tangibly investigate their ideas. However, funding sources can play a role in influencing how researchers conduct and report their study (e.g., scientific bias), which necessitates its disclosure.	All included publications^†^ (*n* = 294)
**Conflict of interest (COI)**		
COI statement: presence of conflict of interest statement	Conflict of interest conveys the authors’ potential associations that may affect the experimental design, methods, and analyses of the outcomes. Thus, full disclosure of possible conflicts allows for unbiased presentation of their study.	All included publications^†^ (*n* = 294)
**Data**		
Data statement: presence of a data availability statement, retrieval method, comprehensibility, and content	Raw data availability facilitates independent verification of research publications. It can improve accountability of outcomes reported and integrity of the research published.	Empirical publications^‡^ (*n* = 195)
**Pre-registration**		
Pre-registration statement: presence of statement indicating registration, retrieval method, accessibility, and contents (hypothesis, methods, analysis plan)	Pre-registration explicitly reports aspects of the study design prior to the commencement of the research. Pre-registration functions as a way to limit selective reporting of results and prevents publication biases and P-hacking.	Empirical publications^‡^ (*n* = 195)
**Protocols**		
Protocol statement: assessed for statement indicating protocol availability, and if available, what aspects of the study are available (hypothesis, methods, analysis plan)	Reproducibility of a study is dependent on the accessibility of the protocol. A protocol is a highly detailed document that contains all aspects of the experimental design which provides a step by step guide in conducting the study.	Empirical publications^‡^ (*n* = 195)
**Analysis scripts**		
Analysis scripts statement: presence of analysis script availability statement, retrieval method, and accessibility	Analysis scripts are used to analyze data obtained in a study through software programs such as R, Python, and MatLab. Analysis scripts provide step by step instructions to reproduce statistical results.	Empirical publications^‡^ (*n* = 195)
**Replication**		
Replication statement: Presence of statement indication a replication study.	Replication studies provide validation to previously done publications by determining whether similar outcomes can be acquired.	Empirical studies^‡^ (*n* = 195)
**Materials**		
Materials statement: presence of a materials availability statement, retrieval method, and accessibility	Materials are the tools used to conduct the study. Lack of materials specification impedes the ability to reproduce a study.	Empirical publications^¶^ (*n* = 191)

The indicators measured for the articles varied depending on its study type. More details about extraction and coding are available here https://osf.io/x24n3/

^†^Excludes publications that have no empirical data

^‡^Excludes case studies and case series

^¶^Excludes meta-analyses and systematic reviews, which materials may not be relevant, in addition to ‡

### Sample characteristics

From our sample of 294 radiology publications, the publishing journals had a median 5-year impact factor of 2.824 (interquartile range 1.765–3.718). Study designs of sampled publications are made available in Table 2. The majority of authors were from the USA (102/294, 34.7%), followed by China (19/294, 6.5%). Humans were the most common test subjects (167/294, 56.8%). Most journals publishing the studies were from the USA (209/294, 71.1%), followed by the UK (44/294, 15.0%). A one-way ANOVA test to compare the effect of country of publication (USA, UK with Ireland, and Europe) on the number of reproducibility indicators demonstrated no significance [*F*(2, 283) = 2.83, *p* = .06]. Nearly half (149/300, 49.7%) of the eligible publications required paywall access. A correlation analysis found that publications that were not freely available through open access contained less indicators of reproducibility (*r* = − .51). More than half of the publications failed to provide a funding statement (176/294, 59.9%). Public funding accounted for 16% (47/294) of analyzed publications. The authors reported having no conflicts of interest (COI) in the majority of publications (156/294, 53.1% vs. 38/294, 12.9%). No COI statement was provided 34.0% (100/294) of the time.

**Table 2:** Characteristics of included publications

Characteristic		Original sample ***N*** (%)		Validation sample ***N*** (%)
**Type of study*N*= 294**	No empirical data	79 (26.9)	**Type of study*N*= 198**	44 (22.2)
	Clinical trial	55 (18.7)		55 (27.8)
	Laboratory	44 (15.0)		43(21.7)
	Chart review	42 (14.3)		12 (6.1)
	Cohort	19 (6.5)		18 (9.1)
	Case study	18 (6.1)		11 (5.6)
	Survey	12 (4.1)		3 (1.5)
	Cost effect	7 (2.4)		4 (2.0)
	Case control	6 (2.0)		1 (0.5)
	Cross-sectional	5 (1.7)		3 (1.5)
	Meta-analysis	4 (1.4)		0 (0.0)
	Case series	2 (0.7)		3 (1.5)
	Multiple	1 (0.3)		1 (0.5)
	Other	0 (0.0)		0 (0.0)
**Test subjects*N*= 294**	Humans	167 (56.8)	**Test subjects*N*= 198**	108 (54.6)
	Neither	116 (39.5)		77 (38.9)
	Animals	11 (3.7)		13 (6.6)
	Both	0 (0)		0 (0)
**Country of journal publication*N*= 294**	US	209 (71.1)	**Country of journal publication*N*= 198**	128 (64.7)
	UK	44 (15.0)		36 (18.2)
	Germany	6 (2.0)		8 (4.04)
	France	5 (1.7)		7 (3.54)
	Japan	3 (1.0)		5 (2.53)
	Canada	3 (1.0)		0 (0.0)
	Italy	2 (0.7)		0 (0.0)
	India	1 (0.3)		0 (0.0)
	Other	21 (7.14)		14 (7.1)
**Country of corresponding author*N*= 294**	US	102 (34.7)	**Country of corresponding author*N*= 198**	74 (37.4)
	China	19 (6.5)		13 (6.6)
	Germany	17 (5.8)		16 (8.1)
	Japan	17 (5.8)		12 (6.1)
	Australia	15 (5.1)		11 (5.6)
	South Korea	14 (4.8)		12 (6.1)
	Turkey	13 (4.4)		3 (1.5)
	Canada	13 (4.4)		3 (1.5)
	UK	9 (3.1)		7 (3.5)
	Netherlands	9 (3.1)		6 (3.0)
	France	8 (2.7)		9 (4.5)
	Switzerland	7 (2.4)		5 (2.5)
	India	5 (1.7)		2 (1.0)
	Italy	5 (1.7)		2 (1.0)
	Spain	3 (1.0)		0 (0.0)
	Unclear	2 (0.7)		0 (0.0)
	Other	36 (12.2)		23 (11.6)
**Open access*N*= 300**	Yes ­ found via open-access button	80 (26.7)	**Open access*N*= 198**	67 (33.8)
	Yes ­ found article via other means	71 (23.7)		23 (11.6)
	Could not access through paywall	149 (49.7)		108 (54.5
**5-year impact factor*N*= 272**	Median	2.824	**5 -year impact factor*N*= 182**	2.89
	1st quartile	1.765		2.02
	3rd quartile	3.718		3.55
	Interquartile range	1.953		1.53
**Most recent impact factor year*N*= 300**	2017	266	**Most recent impact factor year*N*= 200**	0
	2018	10		186
	Not found	24		14
**Most recent impact factor*N*= 276**	Median	2.758	**Most recent impact factor*N*= 186**	2.68
	1st quartile	1.823		1.94
	3rd quartile	3.393		3.79
	Interquartile range	1.57		1.85
**Cited by systematic review or meta-analysis*N*= 211**	No citations	193 (91.5)	**Cited by systematic review or meta-analysis*N*= 151**	132 (87.4)
	A single citation	11 (5.2)		13 (8.6)
	One to five citations	7 (3.3)		6 (4.0)
	Greater than five citations	0 (0)		0 (0)
	Excluded in SR or MA	0 (0)		0 (0)
**Cited by replication study*N*= 211**	No citations	211 (100)	**Cited by replication study*N*= 151**	151 (100)
	A single citation	0 (0)		0 (0)
	One to five citations	0 (0)		0 (0)
	Greater than five citations	0 (0)		0 (0)
	Excluded in SR or MA	0 (0)		0 (0)

### Reproducibility-related characteristics

Table 3 lists the 5 reproducibility indicators. Data availability was reported in 11 publications (11/195, 5.6%), but only 9 (9/11, 81.8%) had accessible data. Complete raw data were located in 0.51% of empirical publications (1/195). A materials’ availability statement was found in 23 publications (23/191, 12.0%), but only 18 (18/23, 78.3%) provided access to materials used in the study. Most publications did not provide a pre-registration statement (8/195, 4.1%) or protocol statement (4/195, 2.1%). Specifics of reproducibility-related characteristics are reported in supplemental Table 4. Among the 195 publications containing empirical data, none provided analysis scripts for the reproduction of statistical results (0/195, 0%). None of the publications reported a replication or novel study (0/195, 0%). Few publications were cited in SR or MA (21/211, 10.0%), with 13 cited a single time, 7 cited between two and five times, and 1 cited more than 5 times. There was no association between the number of times a publication had been cited, and the number of reproducibility indicators (− 0.002). None of the publications were cited in replication studies (0/211, 0%).

**Table 3:** Reproducibility-related characteristics of included publications

Characteristics		***N*** (%)	95% CI
**Open-access*N*= 300**	No	149 (49.7)	44.0–55.3
	Yes	151 (50.3)	21.7–31.7
**Funding*N*= 294**	No funding statement	176 (59.9)	54.3–65.4
	Public	47 (16.0)	11.8–20.1
	No funding received	28 (9.5)	24.9–35.3
	Multiple funding sources	22 (7.5)	4.5–10.5
	Non-profit	7 (2.4)	0.7–4.1
	University	6 (2.0)	0.4–3.6
	Private/industry	6 (2.0)	0.0–1.6
	Hospital	2 (0.7)	0.0–1.6
**Conflict of interest statement*N*= 294**	No conflicts	156 (53.1)	47.4–58.7
	No statement	100 (34.0)	28.7–39.4
	Conflicts	38 (12.9)	9.1–16.7
**Data availability*N*= 195**	No statement	184 (94.4)	91.7–97.0
	Available*	11 (5.6)	3.0–8.3
	Not available	0 (0.0)	[0.0]
**Pre-registration*N*= 195**	No statement	187 (95.9)	[93.7–98.1]
	Available*	7 (3.6)	[1.5–5.7]
	Not available	1 (0.5)	[0–1.3]
**Protocol*N*= 195**	Not available	191 (97.9)	[96.3–99.6]
	Available*	4 (2.1)	[0.4–3.7]
**Analysis scripts*N*= 195**	No statement	195 (100.0)	[100.0]
	Not available	0 (0.0)	[0.0]
	Available	0 (0.0)	[0.0]
Material availability*N*= 191*	No Statement	168 (88.0)	[84.2–91.6]
	Available*	23 (12.0)	[8.4–15.7]
	Not available	0 (0.0)	[0.0]

*CI* confidence interval

*Reproducibility-related characteristics that are available contain further specifications within Table 4

**Table 4:** Specifications of reproducibility-related characteristics of included publications

Characteristic specifications			*N* (%)				
**Data available (*n*= 11)**	Data retrieval	Supplementary information hosted by the journal	10 (90.9)				
		Online third party	1 (9.1)				
		Upon request from authors	0 (0.0)				
	Data accessibility	Yes, data could be accessed and downloaded	9 (81.8)				
		No, data count not be accessed and downloaded	2 (18.2)				
	Data documentation^§^	Yes, data files were clearly documented	8 (88.9)				
		No, data count not be accessed and downloaded	1 (11.1)				
	Raw data availability^§^	No, data files do not contain all raw data	6 (66.7)				
		Unclear if all raw data were available	2 (22.2)				
		Yes, data files contain all raw data	1 (11.1)				
**Materials available (*n*= 23)**	Materials retrieval	Supplementary information hosted by the journal	15 (65.2)				
		Online third party	4 (17.4)				
		In the paper	4 (17.4)				
	Materials accessibility	Yes, materials could be accessed and downloaded	18 (78.3)				
		No, materials not be accessed and downloaded	5 (21.7)				
**Protocol available (*n*= 4)**	Protocol contents	Methods	4 (100.0)				
		Analysis plan	0 (0.0)				
		Hypothesis	0 (0.0)				
**Pre-registration available (*n*= 7)**	Registry	ClinicalTrials.gov	6 (85.7)				
		Brazilian Registry of Clinical Trials	1 (14.3)				
	Pre-registration accessibility	Yes, pre-registration could be accessed	6 (85.7)				
		No, pre-registration could not be accessed	1 (14.3)				
	Pre-registration contents^‖^	Methods	6 (100.0)				
		Hypothesis	2 (33.3)				
		Analysis Plan	0 (0.0)				

^§^Data specifications for documentation and raw data availability are provided if data was made accessible

^‖^Pre-registration specifications for contents are provided if pre-registration was made accessible

### Reproducibility validation sample

Publication characteristics of the validation sample were very similar across all characteristics listed in Table 2. Of the 5 key indicators of reproducibility—availability of data, materials, protocols, analysis scripts, and pre-registration—the results of 2 indicators (availability of protocols and analysis scripts) were reproduced, as they fell within the 95% confidence intervals for the proportions from the original sample. Materials’ availability and pre-registration proportions from the validation sample were lower than what was found in the original sample.

## Discussion

Our cross-sectional investigation found that key transparency and reproducibility-related factors were rare or entirely absent among our sample of publications in the field of radiology. No analyzed publications reported an analysis script, a minority provided access to materials, few were pre-registered, and only one provided raw data. While concerning, our findings are similar to those found in the social science and biomedical literature [7, 12]. Here, we will discuss 2 of the main findings from our study.

One factor that is important for reproducible research is the availability of all raw data. In radiology, clinical data and research data are often stored and processed in online repositories. Picture archives and communication systems, such as Digital Imaging and Communications in Medicine (DICOM), allow researchers to observe data code for details for image acquisition, patient positioning, image depth, and bit depth of an image [13]. For example, the osteoarthritis initiative has available and open-access datasets for testing image analysis algorithms. Furthermore, data sharing in radiology can be difficult as data is often in proprietary formats, too large to upload, or may contain private health information. Picture archiving and communication systems (PACS) have been developed for the purpose of storing and retrieving functional imaging data. Doran et al. have created a software prototype to combine the benefits of clinical and research designs to improve productivity and make data sharing more attainable [14]. Additionally, data sharing is uncommon potentially due to radiology journals lacking structured recommendations. Sardanelli et al. discovered only 1 of 18 general imaging journals had policies requesting data for submission [15]. By improving data sharing in radiology, others have the ability to critically assess the trustworthiness of data analysis and result interpretations [16]. For example, the International Committee of Medical Journal Editors (ICMJE) have established a policy which commends the dissemination of research results and datasets [17–19]. With more than 30 radiology and imaging journals being listed as ICMJE members, the ICMJE could have a substantial influence with the enforcement of this data sharing policy [20]. Journals in adherence with other policies have seen a substantial increase over time in studies with data availability statements [18]. A recent survey by the European Society of Radiology research committee found that 98% of respondents would be interested in sharing data, yet only 23 institutions (34%) had previously shared clinical trial data. From the data shared by these 23 institutions, at least 44 additional original works have been published [21].

A second factor lacking in radiology literature was having detailed analysis scripts publically available. In radiology research, many analytic decisions exist ranging from data management, artificial intelligence algorithms, biomarker identification with validation, and sharing necessary items (data, coding, statistics, protocol) [22–25]. A systematic review of 41 radiomic studies demonstrated 16 failing to report detailed software information, 2 failed to provide image acquisition settings, and 8 lacked detailed descriptions about any preprocessing modifications. These 3 methodological areas are important in radiological imaging studies as they can alter results significantly, thus decreasing the reproducibility of study findings [26]. A recent study by Carp et al. further tested possible variations in radiology imaging analysis by using a combination of 5 pre-processing and 5 modeling decisions for data acquisition in functional magnetic resonance imaging (fMRI). This modification in data collection created almost 7000 unique analytical pathways with varying results [27]. For research findings to be reproducible, a detailed analysis script with explicit software information and methodological decision making is necessary. A strategy to work around such complications is to use public repositories such as GitHub.com to provide the exact coding used to analyze study data. Authors can go one step further and provide their data analysis in a “container” such as docker or singularity, which replicate the study calculations in real time while being applicable to other data sets [28]. Investigators should be encouraged to take notes of analysis coding and scripts as to create a detailed explanation to be published with the study [29]. Triphan et al. provide a good example of providing publically available data analysis in the form of complete Python scripts that reproduce the study findings in real time and can be applied to other datasets [30, 31]. These analysis scripts improve the reproducibility of the study outcomes and will hopefully serve as a guide for future radiology publications to follow [25].

## Implications moving forward

Our sample indicates there is room for improvement for the reporting of reproducibility-related factors in radiologic research. Ninety percent of scientists agree that science is currently experiencing a “reproducibility crisis” [5]. When asked how to improve reproducibility in science, a survey found that 90% of scientists suggested “more robust experimental designs, better statistics, and better mentorship” [5]. Here, we expand on how to implement and accomplish these suggestions. We also briefly discuss the role of artificial intelligence in contributing to research reproducibility.

### More robust reporting

To create transparent and reproducible research, improved reporting is needed. For example, a study found irreproducible publications frequently contain inadequate documentation, reporting of methods, inaccessible protocols, materials, raw datasets, and analysis scripts [7, 12]. We encourage authors to follow reporting guidelines with a non-exhaustive list including Standards for Reporting Diagnostic accuracy studies (STARD for diagnostic accuracy studies) [32], Case Report (CARE for case reports and series) [33], and Guidelines for Reporting Reliability and Agreement Studies (GRRAS for reliability and agreement studies) [34, 35]. In radiology, reliability and agreement studies are prevalent as interobserver agreement between radiologists is measured to identify the potential for errors in treatments or diagnostic imaging [36]. The GRRAS is a 15-item checklist required for study findings to be accurately interpreted and reproduced in reliability and agreement studies. Items such as sample selection, study design, and statistical analysis are often omitted by authors [36–38]. Researchers have had success with using the GRRAS specifically, but reporting guidelines in general provide the framework for studies to be understood by a reader, reproduced by researchers, used by doctors, and included in systematic reviews [37, 39, 40].

### Better statistics

The current reproducibility crisis in part is tied to poor statistics. In psychology, a large-scale reproducibility study found that only 39 of 100 original psychology studies could be successfully replicated [41]. In response, the Association for Psychological Science has pioneered several innovations—such as statistical statcheck programs and statistical advisors—to provide expertise on manuscripts with sophisticated statistics or methodological techniques and to promote reproducibility within psychology [42]. Similar to the field of psychology, a study found that 147 of 157 articles published within radiology journals had statistical errors [43]. Based on these previous findings and our own, it is possible that radiology may be experiencing similar transparency and reproducibility problems and should consider promoting improved statistical practices by using a statistician to assist in the review process. StatReviewer [44]—an automated review of statistical tests and appropriate reporting—additionally may aid peer-reviewers whom are not formally trained to detect relevant statistical errors or detailed methodological errors.

### Better mentorship

Quality research practices are constantly evolving, requiring authors to continually stay up to date, or risk being uninformed. Research mentors should oversee the continual education for graduate students, post-docs, fellows, researchers, and health care providers on areas such as experimental design, statistical techniques, and study methodology. We encourage multi-center collaboration and team science, where cohorts of scientists implement the same research protocols to obtain highly precise and reproducible findings [45–47].

### Artificial intelligence

Artificial intelligence (AI) has become an emerging tool within the field of radiology for physicians and researchers alike. The reproducibility of AI results in research projects is important as more data are being recorded and computed by programs without human intervention (*Gundersen et al. 2017*). Clinical radiology has shown similar increased usage of AI with the advent of algorithms to identify common pathology on chest films with an end goal of being applied to CTs or MRIs. In order for these imaging modalities to become a reality for clinicians, AI must be tested to produce reproducible findings and accurate diagnosis. Reproducibility and generalizability of AI results can be achieved for researchers and clinicians through the use of agreed-upon benchmarking data sets, performance metrics, standard imaging protocols, and reporting formats (*Hosny et al. 2018*).

## Strengths and limitations

Regarding strengths, we randomly sampled a large selection of radiology journals. Our double data extraction methodology was performed in a similar fashion to systematic reviews by following the *Cochrane Handbook* [48]. Complete raw data and all relevant study materials are provided online to ensure transparency and reproducibility. Finally, we extracted data on a second random sample to validate and reproduce our initial results. This validation effort yielded similar results for some indicators and suggests some level of assurance concerning the stability of these estimates across samples. Regarding limitations, our analysis included only 300 of the 53,328 returned publications in the radiology literature; thus, our results may not be generalizable to publications in other time periods outside of our search or medical specialties. Our study focused on analyzing the transparency and reproducibility of the published literature in radiology, and as such, we relied solely on information reported within the publications. Therefore, it cannot be assumed that reproducibility-related factors are not available upon request from the author. Had we contacted the corresponding authors of the 300 analyzed publications, it is plausible we could have obtained more information.

## Conclusion

With the potential lack of transparency and reproducibility practices in radiology, opportunities exist to improve radiology research. Our results indicate important factors for reproducibility and transparency are frequently missing in the field of radiology, leaving room for improvement. Methods to improve reproducibility and transparency are practical and applicable to many research designs.

## Abbreviations

COI: Conflicts of interest
GRRAS: Guidelines for Reporting Reliability and Agreement Studies
ICMJE: International Committee of Medical Journal Editors
ISSN: International Standard Serial Number
NLM: National Library of Medicine
PRISMA: The Preferred Reporting for Systematic Reviews and Meta-Analyses
